# Comparison of the long-term prognostic value of different frailty instruments in older inpatients: a 5-year prospective cohort study

**DOI:** 10.1186/s40001-025-02663-8

**Published:** 2025-05-26

**Authors:** Min Zeng, Yu-Hao Wan, Yao-Dan Liang, Jing Shi, Zhi-Kai Yang, Ting Wang, Chen Ji, Wei He, Ning Sun, Di Guo, Ling-Ling Cui, Lin Yang, Jie-Fu Yang, Hua Wang

**Affiliations:** 1 Department of Cardiology, Beijing Hospital, National Center of Gerontology, Institute of Geriatric Medicine, Chinese Academy of Medical Sciences, Beijing, People’s Republic of China; 2 Beijing Hospital, National Center of Gerontology, Institute of Geriatric Medicine, Chinese Academy of Medical Sciences & Peking Union Medical College, Beijing, People’s Republic of China; 3 Department of Pulmonary and Critical Care Medicine, Beijing Hospital, National Center of Gerontology, Institute of Geriatric Medicine, Chinese Academy of Medical Sciences, Beijing, People’s Republic of China; 4 The Key Laboratory of Geriatrics, Beijing Institute of Geriatrics, Institute of Geriatric Medicine, Chinese Academy of Medical Sciences, Beijing Hospital/National Center of Gerontology of National Health Commission, Beijing, 100730 China

**Keywords:** Frailty scale, All-cause mortality, Long-term prognosis, Older hospitalized patients, Fatigue

## Abstract

**Background:**

Frailty is associated with increased mortality in older adults, but limited studies compare frailty instruments among inpatients with long-term follow-up.

**Aims:**

To evaluate five frailty scales for predicting 5-year all-cause mortality in older inpatients.

**Methods:**

This prospective cohort study enrolled 917 inpatients aged ≥ 65 years. We used five commonly used scales [Clinical Frailty Scale (CFS), FRAIL, Fried, Edmonton, and the comprehensive geriatric assessment-frailty index (CGA-FI)] to screen or assess frailty and then conducted a 5-year telephone follow-up. The primary endpoint was 5-year all-cause mortality. The predictive value of different frailty scales was compared using Kaplan–Meier (K–M) survival analysis, COX regression models, and the receiver operating characteristic (ROC) curves.

**Results:**

The prevalence of frailty ranged from 19.5 to 36.5%. Both K–M survival curves and Cox regression confirmed that frailty patients had higher mortality risk across all scales. After multivariate adjustment, the hazard ratios from highest to lowest, were: CGA-FI, FRAIL, Fried, CFS, and Edmonton (all *p* < 0.05). Frailty demonstrated moderate performance, with area under the curves (AUCs) ranging from 0.70 to 0.75 (all *p* < 0.001). CGA-FI had the largest AUC of 0.724, revealing the best predictive value, while FRAIL had the smallest AUC of 0.666. The AUCs of Fried, Edmonton, and CFS gradually decreased, with no statistical differences. Furthermore, CFS has the highest sensitivity (77.5%).

**Conclusions:**

Frailty identified by all scales is associated with an increased risk of long-term mortality. CFS is the preferred frailty screening scale, while CGA-FI is the most accurate assessment scale.

*Trial registration* ChiCTR1800017204 (07/18/2018).

**Supplementary Information:**

The online version contains supplementary material available at 10.1186/s40001-025-02663-8.

## Introduction

The American Geriatrics Society defined frailty as a non-specific state in which older adults experience increased vulnerability in the body and decreased stress resistance due to a decline in physiological reserves, and susceptibility to a series of adverse clinical events when exposed to relatively small external stressors [1]. The prevalence of frailty in older hospitalized patients is approximately 30% [2] and the global incidence of frailty in community-dwelling older people was estimated to be 43.4 new cases per 1000 person-years [3]. As aging is an independent risk factor for frailty [4], the prevalence of frailty will increase further as the population ages [5]. Frailty increases the risk of serious adverse events, impairs quality of life [6], and poses a significant threat to the health of older adults, becoming a public health event of widespread concern.

Nowadays, various scales are available to assess frailty and show strong associations with all-cause mortality. However, there is no “gold standard” for assessing frailty yet. A meta-analysis reveals that community-dwelling adults defined as frailty by the Fried or FI or FRAIL scale had an all-cause mortality rate 2.4 times higher than healthy adults [7]. Aguayo analyzed the relationship between 35 frailty measurements and all-cause mortality: after adjusting for gender and age, all frailty measurements had significant predictive value for all-cause mortality [8]. Recent studies also support the feasibility and clinical utility of frailty scales in primary care settings, including mobile app-based detection systems and FRAIL-based risk factor analysis [9, 10].

The rationale of this study: previous studies mainly used a single scale, rarely applying multiple scales simultaneously to assess frailty and compare different scales, and the follow-up times generally tend to be short [11]. To the best of our knowledge, no studies compare different frailty scales, in terms of their long-term prognostic value in older hospitalized patients. It is still unclear which frailty scale is most effective and accurate in predicting the long-term mortality risk of older inpatients. In addition, the majority of the published studies are from Western cohorts, primarily focusing on the community-dwelling older population, which may not be directly applicable to Chinese hospitalized patients.

Thus, there is still a lack of studies comparing the long-term prognostic performance of different frailty instruments in older hospitalized patients, particularly in non-Western populations. Understanding which scale most effectively predicts long-term mortality risk is essential for improving risk stratification and guiding clinical decision-making in this vulnerable group. To address this gap, we conducted this 5-year prospective cohort study, selecting the five most commonly used frailty scales[12], to evaluate the prognostic value of different models, with the aim of identifying a frailty scale that more accurately predicts long-term all-cause mortality in hospitalized patients.

## Methods

### Study design

We previously conducted a prospective cohort study of older patients in China (Trial registration: ChiCTR1800017204; registration date: 07/18/2018), consecutively recruiting patients 65 years and older in a tertiary referral hospital (Beijing, China) between September 2018 and February 2019. During hospitalization, we used five scales to screen or assess frailty. Based on the previous cohort study [13], a 5-year telephone follow-up was conducted to collect data on all-cause mortality.

### Participants

Patients recruited were from ten diverse medical or surgical wards (cardiology, respiratory, geriatric, neurology, rehabilitation, traditional Chinese medicine, general surgery, orthopedics, urology, and cardiac surgery). The inclusion criterion was hospitalized patients aged 65 years and above. Exclusion criteria included inability to complete the questionnaire or follow-up (e.g. severe cognitive impairment, deafness, non-Chinese speaking, etc.), expected survival of less than 1 year, and refusal to sign the informed consent.

We screened 1342 older inpatients, excluding 322 patients due to inability to complete the assessment or refusal to sign the informed consent form, and 20 patients did not complete the assessment. Finally, 1000 patients were admitted into the cohort study. During the subsequent 5-year follow-up, 83 patients were progressively lost to follow-up, and 917 patients were ultimately included in the analysis (Supplementary Fig. 1).

### Sample size calculation

Based on previous studies, the prevalence of frailty was 28% [13], and the all-cause mortality rate among frailty individuals was 2.4 times higher than that of robust individuals, with an overall population mortality rate of around 10% [7]. The required sample size was calculated using the ssizeEpi.default() function [14] from the powerSurvEpi package in R (version 4.3.3), assuming a two-sided significance level of 0.05, 80% power and based on the parameters above. The estimated total sample size was 535. After accounting for an anticipated dropout rate of 20%, the adjusted target sample size was approximately 670 participants. Eventually, 917 participants were enrolled, exceeding the estimated requirement and ensuring sufficient statistical power for the survival analyses.

### Information collection

To ensure data accuracy, baseline information was collected through case report forms by trained fixed investigators, including demographics, medical history, comorbidity, physical examination, echocardiographic data, and blood test results. Participants underwent assessments relative to frailty, emotional state, physical function, and cognition immediately after enrollment, which were conducted by experienced nurses. Prior to study initiation, all researchers received standardized training. The study data were compiled and managed using the Research Electronic Data Capture (REDCap) system, with the entire study process supervised by the Clinical Research Institute of Peking University.

### Frailty scales

While numerous tools exist for screening or assessing frailty, there is no recognized gold standard. Frailty scales are largely based on two fundamental concepts: the Frailty Phenotype and the Deficit Accumulation Model [15]. We selected five of the most widely used scales in clinical practice [5]: CFS and FRAIL were used to screen for frailty, whereas Fried, Edmonton, and the CGA-FI were used for frailty assessment. Details of the 5 frailty scales are shown in Supplementary Tables 1–5.

#### *1. The Clinical Frailty Scale *[16]* (CFS, scores between 1 and 9; scores* ≥ *5 defined as frailty)*

CFS is a rapid frailty screening tool, where researchers score based on clinical judgments of subjects’ physical activity level, energy, self-care, and expected lifespan, as well as semi-quantitative assessments of Instrumental Activities of Daily Living (IADL) and Basic Activities of Daily Living (BADL). Namely, subjects will be classified as frailty if they have limited dependence on others for IADL.

#### *2. The FRAIL Scale *[17]* (FRAIL, scores between 0 and 5; scores* ≥ *3 defined as frailty)*

FRAIL is a screening scale in the form of an interview that doesn’t require physical performance measures, therefore it is relatively simple and efficient. FRAIL includes five areas: fatigue, resistance (ability to climb 10 steps without resting), ambulation (ability to walk 100 m), illnesses (greater than 5), and unintentional weight loss (> 5%).

#### *3. The Fried Frailty Phenotype *[18]* (Fried, scores between 0 and 5;meet *≥ *3 criteria defined as frailty)*

The Fried serves as a typical physical frailty criterion and the most basic frailty assessment tool, evaluating from five aspects: unintentional weight loss, exhaustion (fatigue), decreased grip strength, slow walking speed, and reduced physical activity.

#### *4. The Edmonton Frail Scale *[19]* (Edmonton, scores between 0 and 17; scores* ≥ *8 defined as frailty)*

EFS is a validated multidimensional frailty assessment scale consisting of 10 domains: cognition (using the “Clock test”), general health status (number of hospitalizations within a year and self-health attitude), functional independence (using IADL), social support, medication use, nutrition (any recent weight loss), mood (feel sad or depressed), incontinence, and functional performance (using Timed Get Up and Go).

#### *5. The comprehensive geriatric assessment-frailty index (CGA-FI, scores between 0.03 and 0.67; scores* ≥ *0.25 defined as frailty)*

The CGA-FI is a frailty index (FI) based on the comprehensive geriatric assessment (CGA) and Professor Rockwood published a standard procedure for establishing a FI in 2008 [20]. In our previous study, based on the above core criteria, 48 items from multiple domains were selected to construct the CGA-FI, including BADL and IADL, chronic diseases, mood, Mini-Mental State Examination (MMSE), the ability of activity, geriatric syndromes, and some objective physical measures. The CGA-FI score is calculated by dividing the total score of all variables by 48.

### Endpoint and follow-up

The primary endpoint was 5-year all-cause mortality. The follow-up time was calculated from enrollment, and all subjects were followed up by telephone at 3 months, 6 months, and annually (until the fifth year) after enrollment. The follow-up mainly includes survival status (death, time of death, cause of death). When signing the informed consent form, patients provided their phone numbers, as well as those of their cohabitants or relatives. To ensure the accuracy and consistency of the survival status obtained, three trained and specialized nurses conducted telephone follow-ups, and the researchers also checked the patient's hospitalization records and outpatient visit information.

### Statistical analysis

The Kolmogorov–Smirnov tests were used to evaluate whether a continuous variable follows a normal distribution. Normally distributed continuous variables were represented as mean ± standard deviation (SD), and independent samples t-tests were used for intergroup comparison. Non-normally distributed continuous variables were expressed as median [interquartile range: 25 th to 75 th percentiles], and the e Mann–Whitney U-test was performed for intergroup comparison. Categorical variables were described as frequencies (percentages), and comparisons using the Chi-square test or Fisher’s exact test.

K–M methods were utilized to plot the cumulative survival curves of five frailty scales and the log-rank test was used to compare the differences between the two groups. Univariate and multivariate Cox regression models were used to analyze the independent risk factors for 5-year all-cause mortality and the prognostic significance of different frailty scales. The results were presented as hazard ratios (HR) values and 95% confidence intervals (95% CI). Factors from demographics, physical examination, comorbidity, CGA, laboratory, and echocardiographic tests were analyzed individually by univariate COX analysis. Those with a *p* value < 0.05 or clinically meaningful were included in the multivariate COX model for adjustment, thus identifying factors independently associated with all-cause mortality. Model 1 adjusted for age and gender. Model 2 was further adjusted for age, gender, years of education, retirement income, BMI, atrial fibrillation, heart failure, cancer, lg (NT-proBNP), hsCRP, hemoglobin, prealbumin, and creatinine.

ROC curves were used to compare the ability of different frailty scales to distinguish between patients who died and survived after a 5-year follow-up. The AUCs ranged from 0.5 to 1, with larger values indicating superior predictive capacity. The optimal cutoff values were determined using Youden’s index (calculated as sensitivity + specificity −1), with the sensitivity and specificity being calculated at the cutoff values. The DeLong test was used to compare differences in AUCs of five different scales.

Two-tailed *p*-value of less than 0.05 or 0.001 was considered statistically significant. The IBM SPSS Statistics software (version 26) statistical package was used for all calculations, including descriptive statistics, survival analyses (Kaplan–Meier and Cox regression), and ROC curve comparisons (DeLong test). Based on the above analysis, we found that for predicting long-term mortality, CFS is the preferred frailty screening scale (with the highest sensitivity of 0.775), while the CGA-FI (with the largest AUC of 0.724) serves as the most accurate assessment scale. These findings achieve our primary research objectives.

## Results

### Baseline characteristics

During the 5-year follow-up period, 4 patients were lost to follow-up at 3 months, 3 at 6 months, 9 at 1 year, 14 at 2 years, 11 at 3 years, 19 at 4 years, and 23 at 5 years. In total, 83 patients were lost to follow-up, giving an overall loss rate of 8.3% (Supplementary Fig. 1). A total of 917 older hospitalized patients completed the 5-year follow-up, including 422 females (48.2%) and 475 males (51.8%); the average age was 75.3 ± 6.8 years and the oldest was 95. The baseline patient characteristics were largely consistent regardless of the scale applied, with only a few exceptions as shown in Table 1.

**Table 1:** Baseline characteristics between non-frail and frail participants

	CFS		FRAIL		Fried		Edmonton		CGA-FI	
	Non-frail (n = 582)	Frail (n = 335)	Non-frail (n = 738)	Frail (n = 179)	Non-frail (n = 624)	Frail (n = 293)	Non-frail (n = 588)	Frail (n = 329)	Non-frail (n = 684)	Frail (n = 233)
Demographics										
Age, y	73.56 (6.06)***	78.28 (6.87)***	74.54 (6.55)***	78.36 (6.77)***	73.91 (6.36)***	78.22 (6.66)***	74.32 (6.56)***	78.10 (6.57)***	73.31 (5.95)***	78.82 (6.69)***
Gender, male, n (%)	319 (54.8)*	156 (46.6)*	394 (53.4)	81 (45.3)	325 (52.1)	150 (51.2)	378 (55.3)***	97 (41.6)***	329 (56.0)***	146 (44.4)***
Years of education	11.39 (3.95)***	9.96 (4.84)***	11.08 (4.25)**	9.99 (4.68)**	11.17 (4.13)**	10.22 (4.72)**	11.23 (4.04)***	9.80 (5.01)***	11.41 (4.05)***	9.89 (4.69)***
Retirement income (%)										
< 4000	157 (27.4)	100 (30.5)	199 (27.5)	58 (32.6)	171 (27.9)	86 (29.7)	183 (27.2)	74 (32.5)	161 (27.9)	96 (29.6)
4000–8000	321 (55.9)	175 (53.4)	401 (55.4)	95 (53.4)	339 (55.4)	157 (54.1)	375 (55.6)	121 (53.1)	322 (55.7)	174 (53.7)
> 8000	96 (16.7)	53 (16.2)	124 (17.1)	25 (14.0)	102 (16.7)	47 (16.2)	116 (17.2)	33 (14.5)	95 (16.4)	54 (16.7)
Living alone, n (%)	50 (8.6)	30 (9.0)	60 (8.1)	20 (11.2)	51 (8.2)	29 (9.9)	50 (7.3)*	30 (12.9)*	43 (7.3)	37 (11.2)
Physical function and hospitalized events										
Falls^a^, n (%)	136 (23.4)***	119 (35.5)***	189 (25.6)**	66 (36.9)**	147 (23.6)***	108 (36.9)***	152 (22.2)***	103 (44.2)***	113 (19.2)***	142 (43.2)***
Barthel Index	100 [95, 100]***	85 [65, 95]***	100 [90, 100]***	80 [65, 95]***	100 [95, 100]***	85 [65, 95]***	100 [90, 100]***	85 [65, 95]***	100 [95, 100]***	85 [65, 95]***
Hospitalizations^b^	0.37 (0.48)**	0.48 (0.50)**	0.38 (0.48)***	0.55 (0.50)***	0.38 (0.48)**	0.48 (0.50)**	0.33 (0.47)***	0.64 (0.48)***	0.35 (0.48)***	0.51 (0.50)***
Hospital stay, days	7 [5, 11]***	9 [6, 14]***	7 [5, 11]***	10 [7.15]***	7 [5, 11]***	9 [6, 15]***	7 [5, 11]***	9 [6.5, 14]***	7 [5, 10]***	9 [6.14]***
Physical examination										
BMI, kg/m^2^	25.16 (3.31)	24.84 (3.85)	25.12 (3.42)	24.73 (3.91)	25.29 (3.32)**	24.52 (3.87)**	25.10 (3.42)	24.86 (3.81)	25.23 (3.29)*	24.70 (3.88)*
SBP, mmHg	135.14 (16.63)	134.82 (20.44)	135.56 (17.67)	132.82 (19.68)	135.73 (16.69)	133.53 (20.74)	136.01 (17.21)**	132.13 (20.25)**	135.09 (16.67)	134.91 (20.43)
DBP, mmHg	75.97 (9.76)*	74.55 (10.98)*	75.73 (9.83)	74.29 (11.74)	75.85 (9.63)	74.60 (11.40)	76.16 (9.71)***	73.38 (11.44)***	75.82 (9.61)	74.79 (11.27)
Heart Rate, bpm	68.27 (10.36)***	71.67 (12.90)***	68.83 (11.00)***	72.33 (12.85)***	68.45 (10.77)***	71.78 (12.55)***	69.03 (11.06)*	70.93 (12.50)*	68.28 (10.49)***	71.71 (12.75)***
Comorbidity										
Hypertension, n (%)	394 (67.7)***	261 (77.9)***	516 (69.9)*	139 (77.7)*	441 (70.7)	214 (73.0)	478 (69.9)	177 (76.0)	396 (67.3)***	259 (78.7)***
CAD, n (%)	308 (52.9)	183 (54.6)	392 (53.1)	99 (55.3)	332 (53.2)	159 (54.3)	366 (53.5)	125 (53.6)	311 (52.9)	180 (54.7)
AF, n (%)	89 (15.3)	64 (19.1)	116 (15.7)	37 (20.7)	92 (14.7)*	61 (20.8)*	110 (16.1)	43 (18.5)	87 (14.8)*	66 (20.1)*
Heart failure, n (%)	44 (7.6)***	69 (20.6)***	63 (8.5)***	50 (27.9)***	48 (7.7)***	65 (22.2)***	61 (8.9)***	52 (22.3)***	44 (7.5)***	69 (21.0)***
MI, n (%)	47 (8.1)	35 (10.4)	56 (7.6)**	26 (14.5)**	47 (7.5)*	35 (11.9)*	56 (8.2)	26 (11.2)	46 (7.8)	36 (10.9)
Stroke/TIA^c^,n (%)	93 (16.0)***	103 (30.7)***	124 (16.8)***	72 (40.2)***	105 (16.8)***	91 (31.1)***	114 (16.7)***	82 (35.2)***	75 (12.8)***	121 (36.8)***
DM, n (%)	191 (32.8)	118 (35.2)	234 (31.7)*	75 (41.9)*	204 (32.7)	105 (35.8)	211 (30.8)**	98 (42.1)**	173 (29.4)***	136 (41.3)***
Cancer, n (%)	63 (10.8)	40 (11.9)	86 (11.7)	17 (9.5)	68 (10.9)	35 (11.9)	81 (11.8)	22 (9.4)	64 (10.9)	39 (11.9)
Laboratory										
NT-proBNP, pg/mL	126 [60.13, 303.1]***	273.5 [118.85, 893.4]***	147 [67.37, 336.8]***	385.7 [112.85, 1237]***	127.6 [64.1, 300.85]***	306.4 [116.1, 970.7]***	139.2 [66.45, 346.1]***	281.7 [116.6, 815.95]***	127.2 [60.13, 306.2]***	260.3 [117.55, 818]***
hsCRP, mg/L	1.06 [0.58, 2.07]***	1.47 [0.66, 4.91]***	1.05 [0.57, 2.16]***	1.71 [0.86, 7.23]***	1.06 [0.58, 2.07]***	1.54 [0.66, 4.97]***	1.1 [0.59, 2.1]**	1.57 [0.6, 4.75]**	1.01 [0.56, 2.04]***	1.57 [0.70, 4.74]***
Hemoglobin, g/L	129.85 (16.22)***	124.24 (16.38)***	129.48 (15.58)***	120.80 (18.28)***	129.93 (15.60)***	123.25 (17.42)***	129.66 (16.27)***	122.30 (15.95)***	130.24 (16.13)***	123.43 (16.25)***
Albumin, g/L	66.58 (5.26)***	65.35 (5.57)***	66.37 (5.28)**	65.16 (5.84)**	66.54 (5.21)***	65.25 (5.72)***	66.29 (5.39)	65.66 (5.44)	66.39 (5.26)	65.67 (5.64)
Prealbumin,mg/dL	25.13 (5.98)***	22.73 (5.83)***	24.79 (5.91)***	22.17 (6.16)***	25.17 (5.93)***	22.40 (5.82)***	24.78 (5.96)***	22.93 (6.07)***	25.16 (6.07)***	22.64 (5.60)***
Creatinine,umol/L	72.73 (30.76)**	80.56 (46.32)**	73.05 (25.92)***	86.10 (65.26)***	73.16 (30.96)**	80.82 (47.98)**	72.97 (29.32)***	83.30 (53.84)***	72.91 (31.01)**	80.42 (46.35)**
LVEF (%)	62.87 (6.87)***	60.78 (8.12)***	62.76 (6.49)***	59.34 (9.99)***	62.76 (6.54)***	60.67 (8.86)***	62.66 (6.59)***	60.44 (9.24)***	62.97 (6.42)***	60.56 (8.69)***

Values are shown as mean ± standard deviation or median [interquartile range: 25 th to 75 th percentiles] or frequencies (%)

Bold values denote statistically significant differences

*CFS* The Clinical frailty Scale, *FRAIL* The FRAIL scale, *Fried* The Fried frailty Phenotype, *Edmonton* The Edmonton Frail Scale, *CGA-FI* The comprehensive geriatric assessment-frailty index, *BMI* body mass index, *SBP* systolic blood pressure, *DBP* diastolic blood pressure, *CAD* coronary atherosclerotic disease, *AF* atrial fibrillation, *MI* myocardial infarction, including acute and old myocardial infarction, *TIA* transient ischemic attack, *DM* diabetes mellitus, *NT-proBNP* N-terminal pro-B-type natriuretic peptide, *hsCRP* high-sensitive C-reactive protein, *LVEF* left ventricular ejection fraction

^a^Fall = History of falls after age 60

^b^Hospitalizations = History of hospitalization within the past 1 year

^c^Stroke/TIA = History of stroke/transient ischemic attack

^*^p < 0.05, **p < 0.01 and ***p < 0.001 between frail and non-frail identified by the same frailty scale

All five frailty scales revealed that frailty patients were older, had fewer years of education, and larger proportions had a history of falls after age 60 and hospitalization within the past year. Their resting heart rate was faster, the proportion of heart failure and prior stroke/transient ischemic attack was significantly higher, and the Barthel Index, LVEF, hemoglobin, and prealbumin values were lower. Frailty patients had higher hsCRP and creatinine levels, and NT-proBNP levels were 2–3 times higher than those of non-frailty patients. They also tended to have longer hospital lengths. In addition, at least two frailty scales indicated that frailty patients had a higher proportion of women, lower BMI, and diastolic blood pressure, with a more significant prevalence of hypertension, atrial fibrillation, myocardial infarction, and diabetes mellitus.

### Frailty prevalence and 5-year all-cause mortality by different scales

Frailty prevalence were separately 36.5% (CFS), 19.5% (FRAIL), 31.9% (Fried), 25.4% (Edmonton), and 35.8% (CGA-FI). Supplementary Fig. 2a shows the prevalence of frailty. There were a total of 151 deaths during the 5-year follow-up period. In the two screening scales CFS and FRAIL: the mortality rates were 9.6% for non-frailty individuals and 28.3% for frailty individuals screened by the CFS. On the FRAIL scale, they were 11.3% and 37.4% respectively. In the other three assessment scales: the mortality rates for non-frailty and frailty populations assessed by Fried were 9.2% and 31.7% respectively, in Edmonton they were 11.5% and 30.9%, and in the CGA-FI they were 7.9% and 31.6%. Supplementary Fig. 2b illustrates 5-year all-cause mortality rates in patients with or without frailty by different scales.

### Comparing the predictive ability of different frailty scales for 5-year mortality

**Fig. 1:**
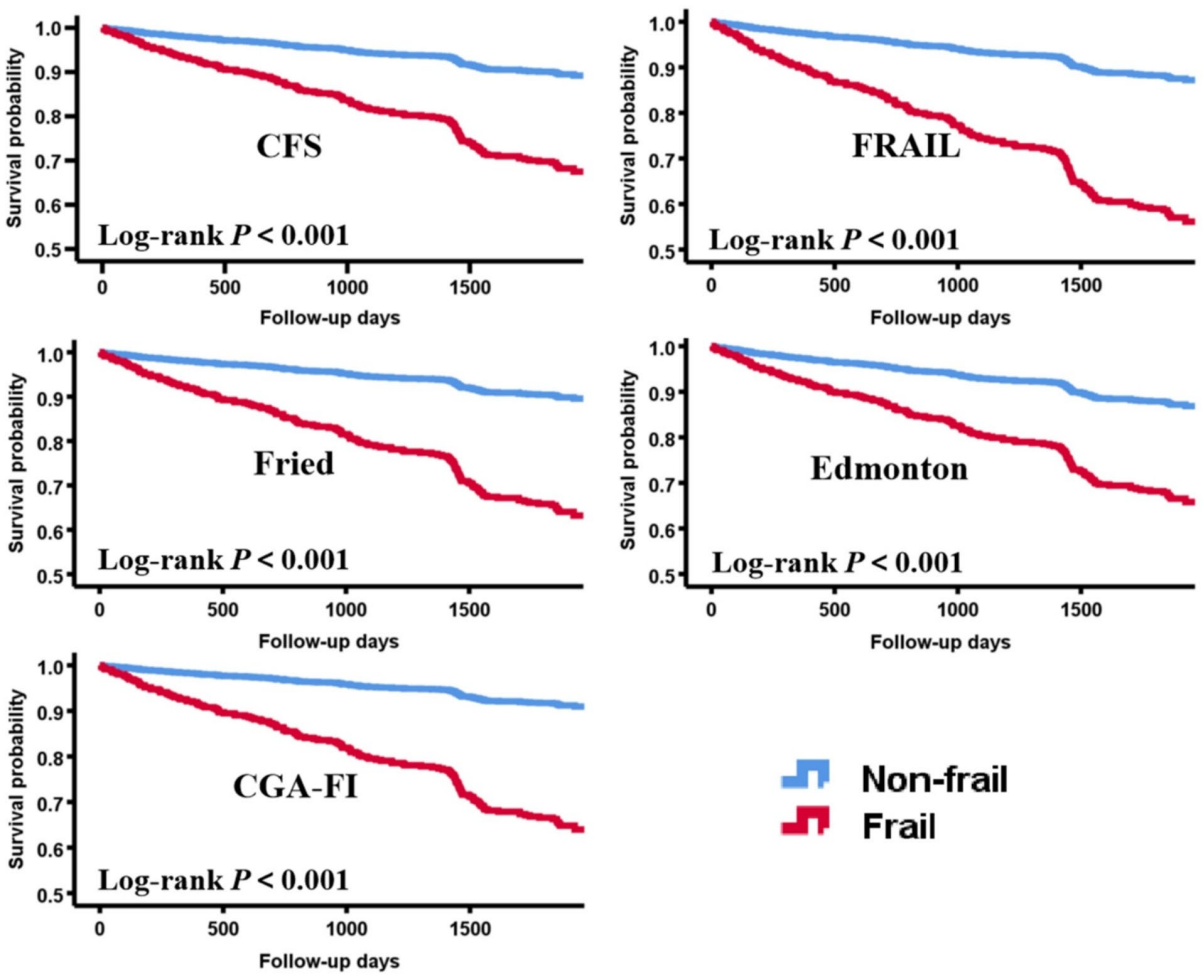
5-Year all-cause mortality Kaplan–Meier curves for different frailty scales

**Fig. 2:**
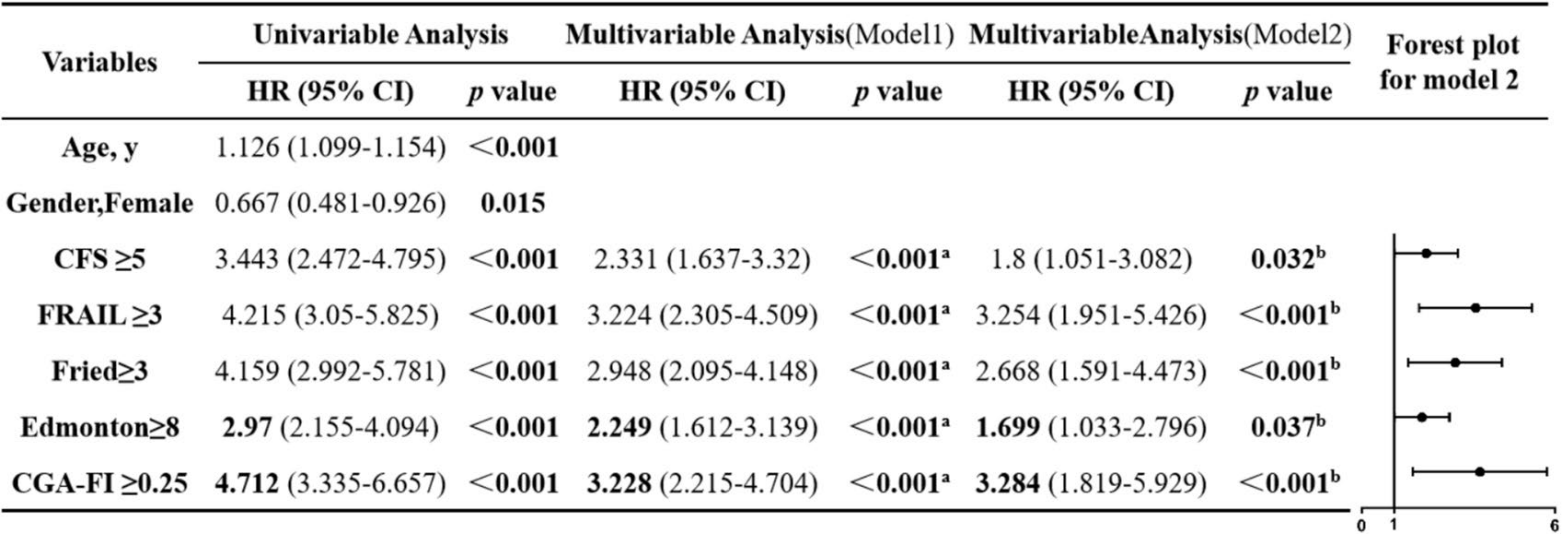
The impact of frailty on 5-year all-cause mortality: univariate and multivariate cox regression analysis with a forest plot. *CI* Confidence interval, *HR* Hazard ratio, *CFS* Clinical Frailty Scale, *FRAIL* FRAIL scale, *Fried* Fried Frailty Phenotype, *Edmonton* Edmonton Frail Scale, *CGA-FI* The comprehensive geriatric assessment-frailty index. Model1 a Respectively adjustment based on age, gender. Model2 b Respectively adjustment based on age, gender, years of education, retirement income, BMI, atrial fibrillation, heart failure, cancer, lg (NT-proBNP), hsCRP, hemoglobin, prealbumin, creatinine

The K–M survival curves demonstrated that frailty defined by each of the five scales was associated with a lower survival rate of all-cause mortality (all log-rank *p* values < 0.001) (Fig. 1). Figure 2 illustrates univariate and two multivariate-adjusted Cox regression models. All three models confirmed that frailty patients had a higher risk of all-cause mortality, regardless of the scale applied. Detailed results are shown in Supplementary Table 6.

The HR for each scale, ranked from highest to lowest, were as follows: CGA-FI, FRAIL, Fried, CFS, Edmonton (Univariate analysis: HR = 4.712, 95%CI 3.335–6.657 for CGA-FI; HR = 4.215, 95%CI 3.05–5.825 for FRAIL; HR = 4.159, 95%CI 2.992–5.781 for Fried; HR = 3.443, 95%CI 2.472–4.795 for CFS and HR = 2.97, 95%CI 2.155–4.094 for Edmonton; Model 2 multivariate analyses (Fig. 2): HR = 3.284, 95%CI 1.819–5.929 for CGA-FI; HR = 3.254, 95%CI 1.951–5.426 for FRAIL; HR = 2.668, 95%CI 1.591–4.473 for Fried; HR = 1.8, 95%CI 1.051–3.082 for CFS and HR = 1.699, 95%CI 1.033–2.796 for Edmonton. All *p*-values for both univariate and multivariate Cox regression were less than 0.05. Multivariable COX regression also revealed that increased age, being male, having a malignancy, high creatinine level, lower levels of hemoglobin and prealbumin are independent risk factors for all-cause mortality within 5 years.

By calculating AUC values, we found that scores of the five scales have a higher predictive value for mortality than their defined frailty status (Table 2). For example, the ROC curve of the CGA-FI based on defined frailty status has an AUC of 0.698 (95% CI 0.651–0.744), whereas the AUC based on the scores is 0.742 (95% CI 0.699–0.786). This was also found in the research by Zhao et al.[21]. Therefore, we recommend using the scores from each frailty scale to predict all-cause mortality, rather than relying solely on whether a patient is classified as frailty.

**Table 2:** AUC and DeLong test of ROC curves for predicting 5-year mortality using different frailty scales

	Frailty or not AUC^a^ (95% CI)	Frailty or not *p*-value	Frailty Score AUC^b^ (95% CI)	Frailty score *p*-value	Cut-off^c^	Sensitivity	Specificity	Score *p*-value^d^ AUC 1 vs. AUC 2, 3, 4, 5	Score *p*-value^d^ AUC 2 vs. AUC 1, 3, 4, 5	Score *p*-value^d^ AUC 3 vs. AUC 1, 2, 4, 5	Score *p*-value^d^ AUC 4 vs. AUC 1, 2, 3, 5	Score *p*-value^d^ AUC 5 vs. AUC 1, 2, 3, 4
1. CFS	0.658 (0.609–0.706)	< 0.001	0.708 (0.663–0.753)	< 0.001	4	0.775	0.557	–	0.052	0.374	0.895	0.015
2. FRAIL	0.649 (0.596–0.701)	< 0.001	0.666 (0.614–0.717)	< 0.001	3	0.444	0.854	0.052	–	< 0.001	0.040	< 0.001
3. Fried	0.677 (0.629–0.726)	< 0.001	0.726 (0.680–0.771)	< 0.001	3	0.616	0.739	0.374	< 0.001	–	0.447	0.364
4. Edmonton	0.633 (0.582–0.685)	< 0.001	0.71 (0.665–0.756)	< 0.001	7	0.629	0.693	0.895	0.04	0.447	–	0.028
5. CGA-FI	0.698 (0.651–0.744)	< 0.001	0.742 (0.699–0.786)	< 0.001	0.25	0.689	0.706	0.015	< 0.001	0.364	0.028	–

*CI* confidence interval, *CFS* The Clinical frailty Scale, *FRAIL* The FRAIL scale, *Fried* The Fried frailty Phenotype, *Edmonton* The Edmonton Frail Scale, *CGA-FI* The comprehensive geriatric assessment-frailty index

^a^Frailty or not AUCs represent the area under the ROC curves plotted based on frailty status

^b^Frailty score AUCs refer to the area under the ROC curves, which are plotted based on the scores of each frailty scale

^c^Cut-off: The cutoff value represents the optimal balance between sensitivity and specificity for the scale’s diagnostic perform

^d^Score *p*-value is obtained by applying the DeLong test to compare the frailty score AUC of one scale with that of others

**Fig. 3:**
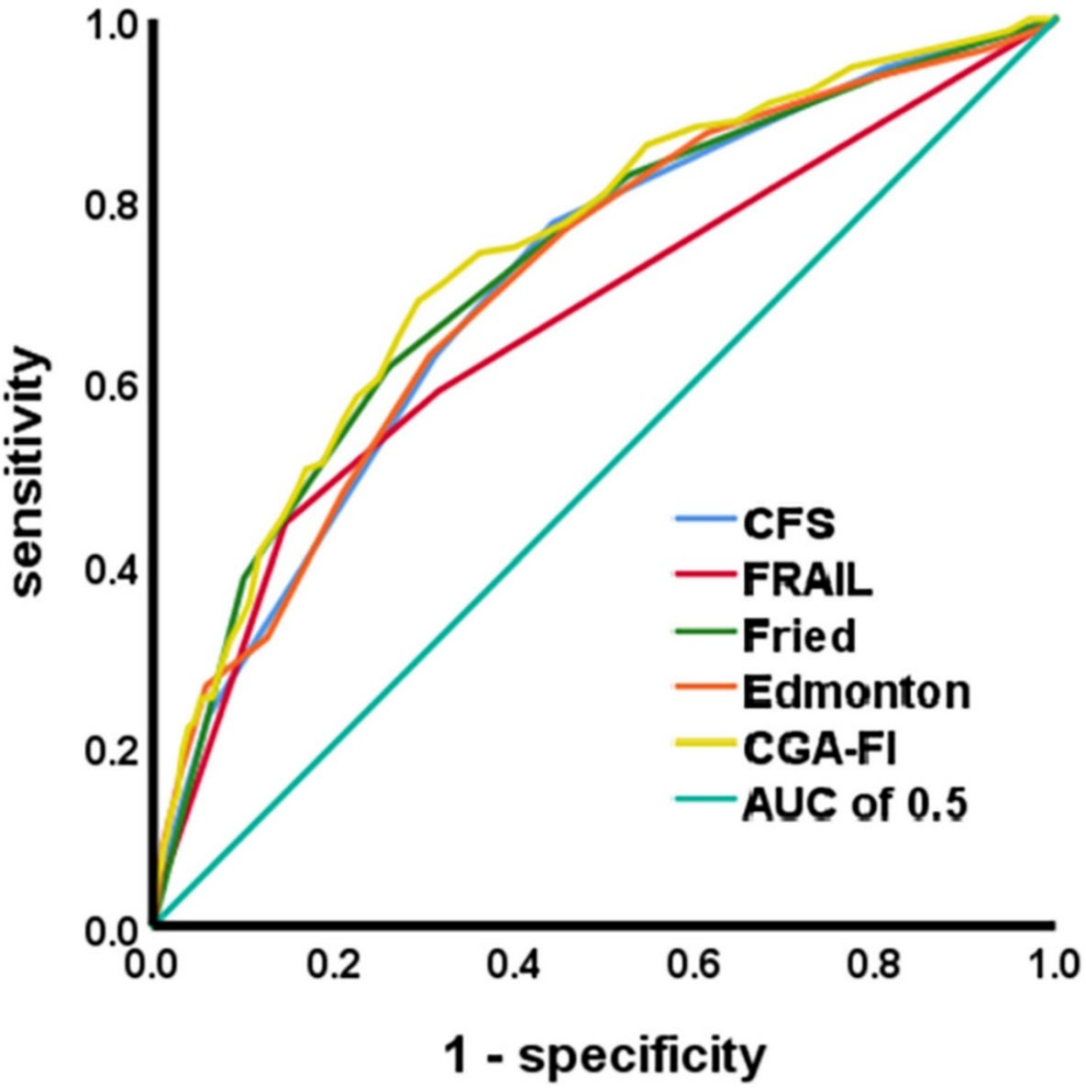
ROC curves: prediction of 5-year mortality using the CFS, FRAIL, Fried, Edmonton and CGA-FI

CFS, FRAIL, Fried, and CGA-FI demonstrated moderate performance in predicting mortality, with AUC values ranging from 0.70 to 0.75, all *p* < 0.001 (Figs. 2, 3). The CGA-FI had the largest AUC of 0.724, and the Delong test revealed statistical differences between it and CFS, FRAIL, and Edmonton, but no statistical difference with Fried (Delong test *p* = 0.015 for CFS, *p* < 0.001 for FRAIL, *p* = 0.028 for Edmonton, and *p* = 0.364 for Fried). The CGA-FI demonstrated the best predictive value and the highest accuracy in predicting 5-year all-cause mortality among hospitalized patients. FRAIL had the smallest AUC of 0.666, which was statistically different from Fried, Edmonton, and CGA-FI (Delong test *p* < 0.001 for Fried and CGA-FI, *p* = 0.04 for Edmonton) and FRAIL was the worst predictor. The AUCs of Fried, Edmonton, and CFS gradually decreased to 0.726, 0.71, and 0.708, respectively, but there were no statistical differences among them (Delong test *p*-values were 0.447 between Fried and Edmonton, 0.374 between Fried and CFS, and 0.895 between Edmonton and CFS). CFS and Edmonton have cutoff values of 4 and 7, respectively, instead of 5 and 8. We also found that CFS had the highest sensitivity at 0.775, making it more suitable for frailty screening.

## Discussion

### The prevalence and harm of frailty

Frailty is prevalent in older adults, with its prevalence varying based on population and assessment methods. Hospitalized patients exhibit a higher prevalence than community-dwelling older adults. We observed that the prevalence of frailty among older inpatients ranged from 19.5 to 36.5%, and in descending order: 19.5% by FRAIL, 25.4% by Edmonton, 31.9% by Fried, 35.8% by CGA-FI, and 36.5% by CFS. O’Caoimh et al. found a frailty prevalence of 30% among hospitalized patients assessed using CGA [2], which is similar to our findings. To some extent, our results more accurately reflect the true frailty prevalence among older inpatients, as we used 5 frailty instruments within the same patient group and enrolled a relatively larger number of participants. Relatively, a large European epidemiologic study applying the harmonized Fried criterion to assess frailty found a prevalence of 7.7% among community-dwelling individuals aged ≥ 50 years [4]. On the other hand, the lack of a gold standard for frailty definition has led to inconsistent epidemiologic reporting. A meta-analysis including 1,755,497 participants reported an overall prevalence of 12% using Fried, compared to 24% in studies using FI [22]. This study also highlighted substantial regional differences in frailty prevalence: using the Fried criteria, the highest prevalence was reported in Africa (22%) and the Americas (17%), while the lowest was observed in Europe (8%). Likewise, FI-based assessments revealed the highest prevalence in Oceania (31%) and the Americas (25%), with Europe again showing the lowest rate (19%) [22]. Another large-scale study further demonstrated that the incidence of frailty and pre-frailty were significantly higher in low- and middle-income countries compared to high-income countries [3], likely due to disparities in socioeconomic status, healthcare access, and comorbidity burden.

Frailty increases the risk of adverse outcomes. As Table 1 demonstrates, frailty patients had a higher rate of falls after age 60, a larger proportion of hospitalizations in the past year, and longer hospital stays. This is consistent with Hoogendijk’s finding that frailty is correlated with increased disability, hospitalization, falls, and healthcare costs [5]. Notably, frailty can be reversed to some extent [23]. Deaths among older adults may be reduced by 3–5% if frailty is prevented or reversed in time [24]. The ability to predict adverse outcomes is the most valued feature of frailty instruments [25], as it helps to identify frailty patients at risk of death as early as possible. This is the initial stride for clinicians to deliver targeted interventions or even reverse frailty and reduce mortality in this vulnerable population. As a result, it is crucial to find a simple, sensitive scale suitable for the preliminary screening of frailty patients at risk of death, as well as a comprehensive and accurate assessment tool that can precisely predict mortality.

### Different frailty scales

As different scales vary significantly in their components and suitability, there is no unified answer regarding which frailty instrument is most suitable for clinical practice. There is a growing consensus that different frailty instruments are preferred for different application settings [26–28] and using a universal approach to assess frailty may not be feasible. When selecting an optimal frailty scale, we need to consider research purposes and the scale’s validity in the relevant application setting.

Frailty scales can be categorized into screening scales and assessment scales based on the time required and intended purposes, though they are often used interchangeably. A frailty screen can be completed in an average of 3 min, while a frailty assessment takes about 15 min and depends on the patient's mobility. Screening scales need to be concise, rapid, highly sensitive, and usually do not need physical tests [26]. This facilitates preliminary frailty screening by clinicians or nurses, allowing for providing appropriate interventions or referral to geriatrics for those who test positive. Assessment scales need to have high specificity, be supported by sound medical theory, accurately predict adverse clinical events such as disability or death, and guide subsequent interventions. Two commonly used screening scales, CFS and FRAIL, and three assessment scales, Fried, Edmonton, and CGA-FI, were selected for this study.

### Assessment scales

Fried provides a complete and accurate measure of physical function and is one of the most commonly used assessment scales in clinical practice. However, it overlooks the effects of disease, cognition, mood, and social support on frailty and has limited use in patients with Parkinson's disease, stroke sequelae, cognitive impairments, and depression. Edmonton, similar to the CGA-FI, is a multidimensional assessment that compensates for Fried’s deficiency of focusing solely on physical frailty. It allows for a more comprehensive assessment of frailty. The reliability and validity of Edmonton have been proven in different populations across several countries [19, 29, 30]. It has been used to assess the occurrence and symptom burden of frailty in heart failure [31], as well as to predict postoperative complications [32]. FI is based on the deficit accumulation model that quantifies the frailty degree by accumulating a series of health deficits, including symptoms, signs, diseases, disabilities, and abnormal laboratory values across multiple domains. CGA is the primary tool used by geriatric specialists to identify frailty in older adults and assess their physiological reserves. The CGA-FI, constructed based on CGA, quantifies the CGA, facilitating a better assessment of the overall health of older individuals. It has been proven to predict various clinical outcomes [33].

Assessment tools are primarily considered for accuracy in predicting adverse events and effectiveness in guiding clinical interventions. The FI is more accurate than the Fried, FRAIL, Groningen Frailty Indicator, Tilburg Frailty Indicator, etc. in predicting short-term outcomes among community-dwelling older adults, although the differences are relatively small [34]. On the other hand, FI is as effective as mortality prediction models (Schonberg index, and Lee index) in predicting 5-year death and provides better predictions for ADL disability and falls. It also offers a cohesive risk stratification method for important health outcomes in older adults [35]. For long-term prognosis: our study found that patients assessed as frailty by CGA-FI had the highest 5-year risk of all-cause mortality (multivariable analysis: HR = 3.284, 95% CI 1.819–5.929). The CGA-FI provided the most accurate prediction (AUC = 0.742, 95% CI 0.699–0.786), significantly outperforming CFS, FRAIL, and Edmonton, as the DeLong test showed a significant statistical difference. This indicates that CGA-FI is superior to Fried and Edmonton as a frailty assessment tool, with better prognostic value. Furthermore, CGA-FI contains multiple potentially modifiable domains suitable for intervention, making it not only predictive but also directly informing the clinical management of patients. For instance, if deficits are concentrated in physical activity, physicians can direct interventions toward physical therapy and rehabilitation under its guidance.

Although the CGA-FI offers a precise assessment of frailty and is useful in predicting mortality and guiding treatment and care plans, obtaining the multidimensional clinical data and physical tests required for a CGA is time-consuming and requires special training for investigators. This makes routine frailty assessment in the clinical frontline still challenging to implement. Therefore, we need to look for frailty screening tools that are more valuable in predicting mortality, take less time, are easier to implement, and are more likely to be applied in busy clinical settings.

### Screening scales

A study designed to evaluate the accuracy of the CFS as a pre-anesthetic frailty screening tool found that the CFS was highly correlated and generally consistent with the assessment scale Edmonton, with comparable effectiveness in identifying frailty [36]. Among older hospitalized patients presenting to the emergency department, it was similarly found that there was no significant difference between CFS and Edmonton in predicting mortality [37]. Sze et al. evaluated frailty in heart failure patients using three screening scales and three assessment scales, finding that CFS showed the strongest correlation and consistency with the three assessment scales, as well as the highest sensitivity [15]. Our study similarly found that CFS had the highest sensitivity (77.5%). In predicting all-cause mortality, the Delong test showed that CFS was comparable to the two frailty assessment scales (Fried and Edmonton), and slightly inferior to the CGA-FI. Another commonly used screening scale, the FRAIL, screens for physical indicators such as fatigue, mobility, and so on. It does not involve cognition or mood, or measure physical performance. FRAIL typically takes about 3 min to complete. In predicting the risk of in-hospital death for older adults admitted due to acute conditions, the FRAIL scale outperformed the FI, Tilburg Frailty Indicator, and CFS. However, the CFS and FI were more effective in predicting 1-year all-cause mortality and readmission [38].

Simplicity and high sensitivity are the primary criteria to consider for screening tools. Our study found that CFS exhibited higher sensitivity compared to FRAIL in investigating whether frailty defined by different scales predicted all-cause mortality (77.5% vs. 44.4%). Other studies have similarly shown that CFS is more sensitive than FRAIL for frailty screening (89.6% vs. 54.6%) [15, 38]. The CFS is time-saving, can be completed in less than 1 min, and has the highest sensitivity. Our study and others have found that CFS is as effective as most lengthy frailty assessments in predicting all-cause mortality and outperforms FRAIL [38]. Therefore, it is highly attractive in clinical practice and we believe the CFS is more suitable for frailty screening than the FRAIL. Promoting CFS in clinical practice is recommended to facilitate the early identification of frailty patients.

### Cutoff values for frailty and pre-frailty

The cutoff value for FI to determine frailty is controversial, and after applying Youden’s index to identify the optimal cutoff, we found that our conclusions are consistent with Song’s study. Specifically, 0.25 is the optimal cutoff for FI in predicting long-term survival [39]. The best cutoff value for the FRAIL, Fried to define frailty is also the same as previous literature, which is 3.

However, we found that the optimal cutoff values were 4 for CFS and 7 for Edmonton (rather than 5 and 8), which are considered to belong to pre-frailty in the literature. Mustafa et al. found that among emergency department patients, the optimal cutoff value for predicting mortality was determined to be 9 for Edmonton and 7 for CFS [37]. These two cutoff values also do not align with previous definitions in the literature. We speculate that the different cutoffs may be related to the context and purpose of use. This reminds us that, when predicting long-term adverse outcomes in older hospitalized patients, it is important to enhance the attention and screening of pre-frailty patients.

### Future directions—electronic frailty index

One of the primary challenges in frailty research is how to apply frailty instruments to clinical practice effectively. To detect frailty patients early, in addition to applying screening scales like CFS, we can also develop the electronic frailty index (eFI).

With the development of big data models, eFI models are continually being developed and improved to cater to various clinical needs [40, 41]: including predicting short-term mortality in heart failure [42], stratifying pre-operative risks [43], and forecasting outcomes in cancers such as multiple myeloma [44] and lung cancer [45]. The eFI does not require additional data collection and significantly reduces the workload for clinicians, making it particularly suitable for low- and middle-income countries where healthcare resources are limited.

In 9315 residents aged 65 and older, the electronic screening index of frailty shows good agreement with mortality, rehospitalization, and health resource consumption [40]. Our previously developed eFI also strongly correlates with the CGA-FI (Pearson’s *r* = 0.716, *p* < 0.001). The eFI ≥ 0.15 is an independent risk factor for prolonged hospitalization and in-hospital mortality, and hospitalization costs increase with the level of frailty [41]. The multimorbidity frailty index (mFI), developed using ICD-10 codes, categorized the study participants according to quartiles: healthy, mild frailty, moderate frailty, and severe frailty. The researchers found that greater frailty was associated with higher risks of 1-year all-cause mortality, unplanned hospitalization, and ICU admission, suggesting that mFI could be used for risk stratification [46]. The validity of the eFI structure [47] was confirmed by Clark et al. These findings make the eFI highly promising and full of potential.

### Study limitations

Firstly, this is a single-center study conducted in Beijing, China, with a limited sample size. Focusing solely on a Chinese population may limit the generalizability of our findings, as the results may vary across different racial, sociocultural or economic contexts. The definition of"older adults"differs internationally: developed countries typically use ≥ 65 years as the threshold, whereas developing countries often adopt ≥ 60 years. The latter group may have disadvantages in educational attainment, economic status, and access to healthcare, which could influence frailty prognosis. External validation is needed for populations in various cities within China or different countries. However, few studies have compared the prognostic impact of different frailty scales, and long-term follow-up studies are even rarer. We included inpatients from different departments, and this study had the largest number of consecutive enrollments, the most frailty scales selected, and the longest follow-up period. Secondly, despite the availability of numerous frailty screening or assessment scales, we only evaluated the 2 frailty screening scales and 3 assessment scales of the most widely used, given the study’s time cost and the participants'cooperation. Finally, we have only compared the predictive value of existing frailty scales for long-term all-cause mortality and do not represent CFS and CGA-FI as the “best” frailty scales.

## Conclusions

Frailty is prevalent among older inpatients, and the prevalence varies using different scales. Frailty defined by CFS, FRAIL, Fried, Edmonton, and CGA-FI are all associated with an increased risk of all-cause mortality. Increased age, being male, having a malignancy, high creatinine levels, lower levels of hemoglobin, and prealbumin are independent risk factors for all-cause mortality within 5 years. Scales showed moderate performance in predicting mortality. The CGA-FI was the most accurate predictor of long-term adverse outcomes among frailty assessment scales, and patients classified as frailty by CGA-FI had the highest 5-year risk of all-cause mortality. The development of eFI contributes to the clinical application of CGA-FI. Among frailty screening scales, CFS is the most sensitive and time-efficient, with accuracy in predicting all-cause mortality comparable to many assessment tools. CFS is more suitable for frailty screening. Widespread use of CFS in clinical practice is recommended for early screening of frailty and pre-frailty patients. The findings are highly useful in geriatric clinical practice and contribute to knowledge regarding frailty screening and assessment scores. It should be noted that this study was conducted in an Eastern (Chinese) population, and the findings may not be directly generalizable to Western populations. Future studies comparing the prognostic performance of frailty instruments across different regions and ethnic groups are warranted.

## Supplementary Information

Supplementary Material 1

## Data Availability

No datasets were generated or analysed during the current study.
