# Supplementary material for: Comparison of the long-term prognostic value of different frailty instruments in older inpatients: a 5-year prospective cohort study
Source: Eur J Med Res. 2025 May 26;30:417. doi: 10.1186/s40001-025-02663-8 (PMC12105130; doi:10.1186/s40001-025-02663-8)
Supplement: Supplementary file 1 — Supplementary Material 1 [file 40001_2025_2663_MOESM1_ESM.pdf]

Supplemental Material

Supplementary Figure 1. Flow Chart of the Frailty Cohort.

Supplementary Figure 2. Frailty Prevalence and 5-Year All-Cause Mortality Rates of Different Scales

Supplementary Table 1. Criteria of Clinical Frailty Scale

Supplementary Table 2. Criteria of FRAIL Scale

Supplementary Table 3. Criteria of Fried Frailty Phenotype

Supplementary Table 4. The Edmonton Frailty Scale

Supplementary Table 5. Factors of Comprehensive Geriatric Assessment - Frailty Index (CGA-FI)

Supplementary Table 6. The Influence of Frailty on 5-Year All-Cause Mortality as Evaluated by the Univariate and Multivariate Cox Regression Model.

Supplementary Figure 1. Flow Chart of the Frailty Cohort.

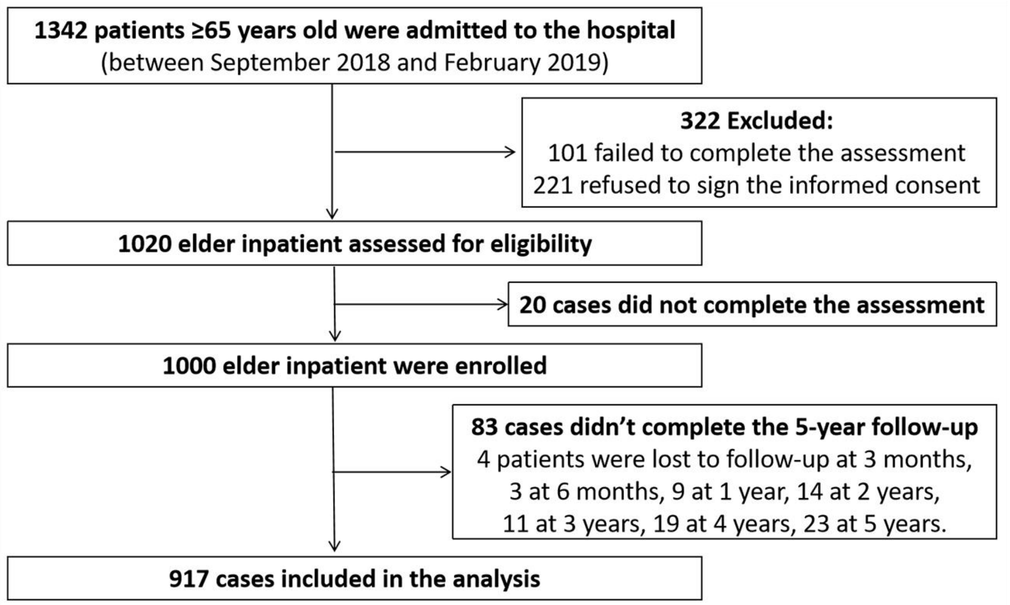

Supplementary Figure 2. Frailty Prevalence and 5-Year All-Cause Mortality Rates of Different Scales

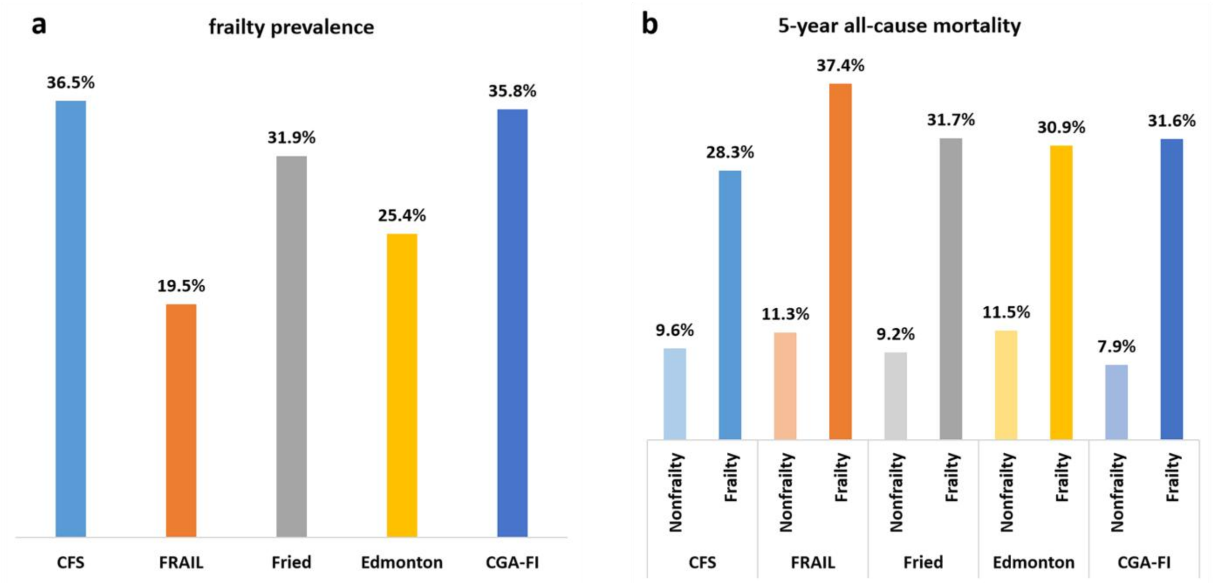

Note: **(a)** Frailty Prevalence of Different Scales

(b) 5-Year All-Cause Mortality Rates in Patients With or Without Frailty by Different Scales

Supplementary Table 1. Criteria of Clinical Frailty Scale

Score	Category	
1	**Very Fit**	People who are robust, active, energetic and motivated. These people commonly exercise regularly. They are among the fittest for their age.
2	**Well**	People who have no active disease symptoms but are less fit than category I. Often, they exercise or are very active occasionally, e.g. seasonally.
3	**Managing Well**	People whose medical problems are well controlled, but are not regularly active beyond routine walking.
4	**Vulnerable**	While not dependent on others for daily help, often symptoms limit activities. A common complaint is being "slowed up", and/or being tired during the day.
5	**Mildly Frail**	These people often have more evident slowing, and need help in high order IADLs (finances, transportation, heavy housework, medications).Typically, mild frailty progressively impairs shopping and walking outside alone, meal preparation and house work.
6	**Moderately Frail**	People need help with all outside activities and with keeping house. Inside, they often have problems with stairs and need help with bathing and might need minimal assistance (cuing, standby) with dressing
7	**Severely Frail**	Completely dependent for personal care, from whatever cause(physical or cognitive). Even so, they seem stable and not at high risk of dying(within ~ 6 months)
8	**Very Severely Frail**	Completely dependent, approaching the end of lifely. Typically, they could not recover even from a minor illness.
9	**Terminally Ill**	Approaching the end of life. This category applies to people with a life expectancy < 6 months, who are not otherwise evidently frail.
1-2= Non-frail ;3-4 = Prefrail ; and ≥5 = Frail		

Supplementary Table 2. Criteria of FRAIL Scale

	Inquiry	Score
**Fatigue**	"How much of the time during the past 4 weeks did you feel tired?"	1=All of the time, 2 =Most of the time, 3=Some of the time, 4=A little of the time, 5 = None of the time. Responses of "1" or "2" are scored as 1 and all others as 0.
**Resistance**	"By yourself and not using aids, do you have any difficulty walking up 10 steps without resting?"	1 = Yes. 0 = No
**Ambulation**	"By yourself and not using aids, do you have any difficulty walking several hundred yards?"	1 = Yes, 0 = No.
**Illness**	"Did a doctor ever tell you that you have [illness]? The illnesses include hypertension, diabetes, cancer(other than a minor skin cancer), chronic lung disease, heart attack, congestive heart failure, angina, asthma, arthritis, stroke, and kidney disease	The total illnesses (0-11) are recoded as 0-4 = 0 and 5-11 = 1
**Loss of weight**	"How much do you weigh with your clothes on but without shoes? (current weight) " "One year ago, how much did you weigh without your shoes and with your clothes on?(weight 1 year ago)"	·Percent weight change is computed as: [(weight 1 year ago - current weight)/weight 1 year ago] x 100% ·Percent change > 5% (representing a 5% loss of weight) is scored as 1 and < 5% as 0
Sum the scores for the 5 questions above.		
0 = Non-frail ; 1-2 = Prefrail ; and ≥3 = Frail		

Supplementary Table 3. Criteria of Fried Frailty Phenotype

	Male	Female
**Unintentional weight loss**	>4.5kg over past year or >5% of previous year's body weight	
**Exhaustion (fatigue)**	Any question ≥3 days / week	
	1. How often in the last week did you feel that you could not get going?	
	2. How often in the last week did you feel like everything you did was an effort?	
**Decreased grip strength**	Dominant hand grip strength measured by CAMRY electronic hand dynamometer for two times average	
	BMI≤24kg/m^2, ≤29kg	BMI≤23kg/m^2, ≤17kg
	24<BMI≤28kg/m^2, ≤30kg	23<BMI≤26kg/m^2, ≤17.3kg
	BMI＞28kg/m^2, ≤32kg	26＜BMI≤29kg/m^2, ≤18kg
		BMI＞29kg/m^2, ≤21kg
**Slow walking speed**	The faster speed to walk 4 meters for two times, with or without a walking aid	
	Height≤173cm, ≥6.1s	Height≤159cm, ≥6.1s
	Height＞173cm, ≥5.3s	Height＞159cm, ≥5.3s
**Reduced physical Activity**	Used the short version of the Minnesota Leisure Time Activity questionnaire	
	<383kcal/week (walking 2.5h/week)	<270kcal/week (walking 2h/week)
0 = Non-frail ; 1-2 = Prefrail ; and ≥3 = Frail		

Abbreviations: BMI=body mass index

Supplementary Table 4. The Edmonton Frailty Scale

Frailty domain	Item	0 point	1 point	2 points
**Cognition**	Please imagine that this pre-drawn circle is a clock and place the numbers in the correct position, then place the hands to indicate a time of 'ten after eleven'	No errors	Minor spacing errors	Other errors
**General health status**	In the past year, how many times have you been admitted to hospital?	0	1-2	≥2
	In general, how would you describe your health?	Excellent/ very good/ good	Fair	Poor
**Functional dependence**	With how many of the following activities do you require help? (meal preparation, shopping, transportation, telephone, housekeeping, laundry, managing money, taking medications)	0-1	2-4	5-8
**Social support**	When you need help, can you count on someone who is willing and able to meet your needs?	Always	Sometimes	Never
**Medication use**	Do you use five or more different prescription medications on a regular basis?	No	Yes	
	At times, do you forget to take your prescription medications?	No	Yes	
**Nutrition**	Have you recently lost weight such that your clothing has become looser?	No	Yes	
**Mood**	Do you often feel sad or depressed?	No	Yes	
**Continence**	Do you have a problem with losing control of urine when you don't want to?	No	Yes	
**Functional performance**	Please sit on this chair with your back and arms resting. Then, when I say 'GO', please stand up and walk at a safe and comfortable pace to the mark on the floor (3meter away) and return to the chair and sit down.	0-10sec	11-20 sec	One of>20sec patient unwilling/ require assistance
**Total**	Final score is the sum of column totals	0-5 = not frail<br>6-7 = vulnerable<br>8-9 = mild frailty<br>10-11 = moderate frailty<br>12-17 = severe frailty		

Supplementary Table 5. Factors of Comprehensive Geriatric Assessment - Frailty Index (CGA-FI)

Frailty index	Cut-off
1.Need help bathing	Yes=1, No=0
2. Need help dressing	Yes=1, No=0
3. Need help using the toilet	Yes=1, No=0
4. Need help getting in / out of chair	Yes=1, No=0
5. Need help feeding	Yes=1, No=0
6. Incontinence	Yes=1, No=0
7. Need help shopping	Yes=1, No=0
8. Need help with finances	Yes=1, No=0
9. Need help using transportation	Yes=1, No=0
10. Need help using telephone	Yes=1, No=0
11. Need help managing medications	Yes=1, No=0
12. Need help with housekeeping	Yes=1, No=0
13. Need help preparing meals	Yes=1, No=0
14. Need help washing clothes	Yes=1, No=0
15. Hypertension	Yes=1, No=0
16. Coronary heart disease	Yes=1, No=0
17. Atrial fibrillation	Yes=1, No=0
18. Congestive heart failure	Yes=1, No=0
19. Peripheral artery disease	Yes=1, No=0
20. Chronic Lung disease	Yes=1, No=0
21. Sleep apnea hypopnea syndrome	Yes=1, No=0
22. Diabetes	Yes=1, No=0
23. Thyroid dysfunction	Yes=1, No=0
24. Peptic ulcer	Yes=1, No=0
25. Stroke	Yes=1, No=0
26. Chronic kidney disease	Yes=1, No=0
27. Osteoarthritis	Yes=1, No=0
28. Osteoporosis	Yes=1, No=0
29. Parkinson’s disease	Yes=1, No=0
30. Cancer	Yes=1, No=0
31. Depression	Yes=1(GDS-5items≥2), No=0(GDS-5items<2)
32. Anxiety	Yes=1(HADS-A≥8), No=0(HADS-A<8)
33. Loneliness	Yes=1, No=0
34. Cognition	MMSE<18 =1; 18≤MMSE<24 =0.5; MMSE≥ 24 =0
35. Visual impairment	Yes=1, No=0
36. Hearing impairment	Yes=1, No=0
37. Chewing impairment	Yes=1, No=0
38. Fall history	Yes=1, No=0

Supplementary Table 5. Factors of Comprehensive Geriatric Assessment - Frailty Index (CGA-FI)

39. Chronic constipation	Yes=1, No=0
40. Chronic pain	Yes=1, No=0
41. Insomnia	Yes=1(AIS≥6), No=0(AIS<6)
42. Depend on assistive devices	Yes=1, No=0
43. Take exercise outside	Yes=1, No=0
44. Body mass index (BMI) (kg/m^2)	BMI≤18 or BMI≥28 =1; 25≤BMI<28 =0.5; 18<BMI<25 =0
45. Calf circumference (cm)	≤25th percentile =1;<br>25th to 50th percentiles =0.5;<br>>50th percentile =0
46. Peak flow (liters/min)	
47. Grip strength (kg)	
48. 4m-walking speed (m/s)	

Abbreviations: AIS=Athens Insomnia Scale; CGA-FI=comprehensive geriatric assessment-frailty index; GDS-5items=5-item Geriatric Depression Scale; HADS-A=Hospital Anxiety and Depression Scale-Anxiety; MMSE=Mini-Mental State Examination

Supplementary Table 6. The Influence of Frailty on 5-Year All-Cause Mortality as Evaluated by the Univariate and Multivariate Cox Regression Model.

Variables	Univariable Analysis				Multivariable Analysis(Model1)				Multivariable Analysis(Model2)			
	HR	95% CI		*P* value	HR	95% CI		*P* value	HR	95% CI		*P* value
		Lower	Upper			Lower	Upper			Lower	Upper	
Age, y	1.126	1.099	1.154	**＜0.001**	1.101	1.074	1.13	**＜0.001**	1.11	1.063	1.16	**＜0.001**
Gender,Ffemale	0.667	0.481	0.926	**0.015**	0.609	0.438	0.846	**0.003**	0.536	0.32	0.899	**0.018**
Years of education,y	0.956	0.922	0.992	**0.016**	-	-	-	-	0.949	0.895	1.005	0.074
Retirement Income[a]	1.283	1.006	1.637	**0.044**	-	-	-	-	0.801	0.527	1.218	0.3
Living alone	1.102	0.636	1.911	0.728	-	-	-	-	-	-	-	-
Falls[b]	1.405	1.002	1.970	**0.049**	-	-	-	-	-	-	-	-
Barthel Index	0.978	0.972	0.984	**＜0.001**	-	-	-	-	-	-	-	-
Hospitalizations[c]	1.67	1.212	2.301	**0.002**	-	-	-	-	-	-	-	-
BMI, kg/m^2	0.906	0.865	0.949	**＜0.001**	-	-	-	-	1.007	0.944	1.075	0.822
Heart Rate, bpm	1.019	1.007	1.032	**0.003**	-	-	-	-	-	-	-	-
Hypertension	1.505	1.023	2.215	**0.038**	-	-	-	-	-	-	-	-
Atrial Fibrillation	1.630	1.116	2.379	**0.011**	-	-	-	-	1.423	0.834	2.428	0.196
Heart failure	3.335	2.327	4.780	**＜0.001**	-	-	-	-	1.109	0.562	2.189	0.766
Myocardial Infarction[d]	2.274	1.469	3.519	**＜0.001**	-	-	-	-	-	-	-	-
Cancer	2.943	2.015	4.298	**＜0.001**	-	-	-	-	3.248	1.801	5.859	**＜0.001**
LVEF,%	0.955	0.939	0.971	**＜0.001**	-	-	-	-	-	-	-	-
lg(NT-proBNP), pg/mL	3.389	2.474	4.642	**＜0.001**	-	-	-	-	1.327	0.765	2.305	0.314
hsCRP, mg/L	1.014	1.008	1.020	**＜0.001**	-	-	-	-	1.01	0.999	1.021	0.062
hemoglobin, g/L	0.971	0.963	0.980	**＜0.001**	-	-	-	-	0.981	0.966	0.997	**0.018**
Prealbumin,mg/dL	0.864	0.831	0.898	**＜0.001**	-	-	-	-	0.927	0.883	0.973	**0.002**
Creatinine, umol/L	1.006	1.004	1.007	**＜0.001**	-	-	-	-	1.005	1.002	1.008	**0.003**

Supplementary Table 6. The Influence of Frailty on 5-Year All-Cause Mortality as Evaluated by the Univariate and Multivariate Cox Regression Model.

Variables	Univariable Analysis				Multivariable Analysis(Model1)				Multivariable Analysis(Model2)			
	HR	95% CI		*P* value	HR	95% CI		*P* value	HR	95% CI		*P* value
		Lower	Upper			Lower	Upper			Lower	Upper	
BADL	0.730	0.663	0.805	**＜0.001**	-	-	-	-	-	-	-	-
MMSE	0.888	0.866	0.910	**＜0.001**	-	-	-	-	-	-	-	-
**Frailty Scales**												
CFS ≥5	3.443	2.472	4.795	**＜0.001**	2.331	1.637	3.32	**＜0.001%**	1.8	1.051	3.082	**0.032#**
FRAIL ≥3	4.215	3.05	5.825	**＜0.001**	3.224	2.305	4.509	**＜0.001%**	3.254	1.951	5.426	**＜0.001#**
Fried≥3	4.159	2.992	5.781	**＜0.001**	2.948	2.095	4.148	**＜0.001%**	2.668	1.591	4.473	**＜0.001#**
Edmonton≥8	**2.97**	2.155	4.094	**＜0.001**	2.249	1.612	3.139	**＜0.001%**	1.699	1.033	2.796	**0.037#**
CGA-FI ≥0.25	**4.712**	3.335	6.657	**＜0.001**	3.228	2.215	4.704	**＜0.001%**	3.284	1.819	5.929	**＜0.001#**

Notes:CI=Confidence Interval; HR=Hazard Ratio; BMI=body mass index;LVEF=left ventricular ejection fraction; hsCRP=high-sensitive C-reactive protein; BADL=Basic Activities of Daily Living; MMSE=Mini-Mental State Examination

[a]Retirement Income: (RMB/Month) <4000=1,4000-8000=2,≥8000=3; [b]Fall=History of falls after age 60; [c]Hospitalizations=History of hospitalization within the past 1 year; [d]Myocardial Infarction: including acute and old myocardial infarction

Model1 % Respectively adjustment based on age, gender

Model2 # Respectively adjustment based on age, gender, years of education, retirement income, BMI, atrial fibrillation, heart failure, cancer, lg(NT-proBNP), hsCRP, hemoglobin, prealbumin, creatinine
